# Comparison of Cervical Spine Injury Clinical Prediction Rules for Children After Blunt Trauma

**DOI:** 10.1001/jamanetworkopen.2025.49403

**Published:** 2025-12-19

**Authors:** Lois K. Lee, Fahd A. Ahmad, Lorin R. Browne, Monica Harding, Lawrence Cook, Kathleen Adelgais, Rebecca K. Burger, Alexander Rogers, Leah Tzimenatos, Lauren C. Riney, Daniel Rubalcava, Caleb E. Ward, Kenneth Yen, Nathan Kuppermann, Julie C. Leonard

**Affiliations:** 1 Division of Emergency Medicine, Department of Pediatrics, Harvard Medical School, Boston Children’s Hospital, Boston, Massachusetts; 2 Department of Pediatrics, Division of Emergency Medicine, Washington University School of Medicine in St Louis, St Louis, Missouri; 3 Division of Pediatric Emergency Medicine, Medical College of Wisconsin, Milwaukee; 4 Utah Data Coordinating Center, University of Utah, Salt Lake City; 5 Section of Pediatric Emergency Medicine, Department of Pediatrics, University of Colorado School of Medicine, Aurora; 6 Department of Pediatrics, Division of Emergency Medicine, Emory University School of Medicine, Children’s Healthcare of Atlanta, Atlanta, Georgia; 7 Departments of Emergency Medicine and Pediatrics, University of Michigan, Ann Arbor; 8 Department of Emergency Medicine, University of California, Davis School of Medicine, Sacramento; 9 Division of Emergency Medicine, Cincinnati Children’s Hospital Medical Center, Cincinnati, Ohio; 10 Division of Pediatric Emergency Medicine, Department of Pediatrics, Baylor College of Medicine, Texas Children’s Hospital, Houston; 11 Division of Emergency Medicine, Department of Pediatrics, The George Washington University School of Medicine and Health Sciences, Washington, DC; 12 Division of Pediatric Emergency Medicine, Department of Pediatrics, Children’s Health Dallas, University of Texas Southwestern Medical Center, Dallas; 13 Division of Emergency Medicine, Department of Pediatrics, The Ohio State University College of Medicine and Nationwide Children’s Hospital, Columbus; 14 Department of Pediatrics, Division of Pediatric Emergency Medicine, University of Texas Southwestern Medical Center, Dallas

## Abstract

**Importance:**

Pediatric cervical spine injury (CSI) is a rare occurrence; however, CSI can result in significant disability and death. It is essential to determine the optimal CSI clinical prediction rule to risk stratify children with potential CSI after blunt trauma who require cervical spine imaging.

**Objective:**

To compare the test characteristics and projected imaging rates between 3 prospectively derived CSI clinical prediction rules: the Pediatric Emergency Care Applied Research Network CSI prediction rule (PECARN CSI rule), the National Emergency X-Radiography Utilization Study (NEXUS), and the Canadian Cervical Spine (c-spine) rule (CCR).

**Design, Setting, and Participants:**

This comparative effectiveness study was a planned secondary analysis of a prospective observational study enrolling from December 2018 to October 2021 in 18 PECARN emergency departments. Eligible participants were children up through age 17 years presenting after blunt trauma. Data were analyzed between March 2024 and January 2025.

**Exposures:**

Enrollment in the primary study to develop and validate the PECARN CSI prediction rule.

**Main Outcome Measures:**

Test characteristics with 95% CIs (sensitivity, specificity, positive predictive value [PPV], negative predictive value [NPV]) and area under the curve (AUC) for the receiver operator curves (ROC) for the detection of CSI using each of the 3 rules. We also estimated the projected c-spine imaging rate (radiography or computed tomography [CT]) based on criteria for each of the 3 rules.

**Results:**

There were 22 430 eligible children enrolled (median [IQR] age 8 [2.0-13.0] years; 13 068 male [58.3%]) and 433 (1.9%) had CSI. C-spine imaging was performed 12 768 children (56.9%): 8912 (39.7%) had radiography and 3856 (17.2%) were imaged with CT. The sensitivity of the 3 rules was: PECARN CSI rule, 93.3% (95% CI, 90.9%-95.7%); NEXUS, 85.7% (95% CI, 82.4%-89.0%); and CCR, 90.8% (95% CI, 88.0%-93.5%). The NPV of the 3 rules was: PECARN, 99.8% (95% CI, 99.7%-99.9%); NEXUS, 99.6% (95% CI, 99.5%-99.7%); and CCR, 99.7% (95% CI, 99.6%-99.8%). Strictly applying each rule resulted in projected CT imaging of 1549 children (6.9%) for PECARN, 2419 (10.8%) for NEXUS, and 2968 (13.2%) for CCR.

**Conclusions and Relevance:**

In this comparative effectiveness study of CSI prediction rules in children, the PECARN CSI rule had the highest sensitivity and NPV for identifying children at risk for CSI after blunt trauma, with the lowest projected CT imaging rate.

## Key Points

**Question:**

Which of 3 prospectively derived clinical cervical spine injury (CSI) prediction rules for radiologic imaging (Pediatric Emergency Care Applied Research Network CSI prediction rule [PECARN CSI rule], National Emergency X-Radiography Utilization Study [NEXUS], Canadian Cervical Spine [c-spine] rule [CCR]) has the best test characteristics?

**Findings:**

In this comparative effectiveness study of the performance of CSI prediction rules in a cohort of 22 430 children, the PECARN CSI rule had the best test characteristics and lowest projected CT imaging rate.

**Meaning:**

The PECARN CSI rule had the best test characteristics for risk stratifying children with potential CSI after blunt trauma who require cervical spine imaging.

## Teaser

This comparative effectiveness study evaluates several clinical cervical spine injury prediction tools for test characteristics and lowest projected computed tomography rate in a secondary analysis of a large cohort of US children.

## Introduction

Pediatric cervical spine injury (CSI) is a rare occurrence, with an estimated prevalence of less 1% to 2% in children presenting after trauma.^1,2,3,4^ Although infrequent, CSI can result in significant long-term disability and death.^5^ Identifying all CSIs in children presenting after trauma is important to prevent complications from missed diagnoses.^6,7,8^ In the emergency department (ED), this is done with the use of imaging, including radiography, computed tomography (CT), and magnetic resonance imaging (MRI).^6^ Given the increased lifetime risks of cancer associated with exposure to ionizing radiation from CT^9,10,11^ and concerns about prolonged spinal motion restriction,^12^ it is essential to identify those children who are at higher risk for CSI and require imaging for cervical spine evaluation, as well as those at low risk for CSI who do not.^2,6,8,13^

Several clinical prediction rules have been developed to assist the clinician in identifying patients at higher risk for clinically important CSI after trauma.^14^ Two widely used prediction rules are the National Emergency X-Radiography Utilization Study Low Risk Criteria (NEXUS)^15^ and the Canadian Cervical Spine (c-spine) Rule (CCR).^16^ The NEXUS rule includes only clinical parameters,^15^ while the CCR also considers the mechanism of injury.^14,16^ These studies were designed to risk stratify adult trauma patients to determine the need for diagnostic c-spine imaging and were not specifically focused on children. The NEXUS study included only 3065 children (9.0%) under age 18 years out of the 34 069 individuals in the study.^17^ The CCR excluded children under age 16 years in their study.^16^ Nonetheless, these rules are used for clinical decision-making in the evaluation of pediatric patients with blunt trauma. Only 1 study, from the Pediatric Emergency Care Applied Research Network (PECARN), has prospectively derived a pediatric CSI rule using a study population of 22 430 children from birth to 17 years. It derived and validated a CSI clinical prediction rule and created a clinical algorithm for cervical spine imaging in children presenting with blunt trauma.^3^ Unlike NEXUS and CCR, which only designate a patient to be at increased risk of CSI,^15,16^ this PECARN rule provides 3 levels of risk to correlate with imaging decisions (clinical clearance [no imaging], radiography, CT).^3^

Given the widespread use of NEXUS and CCR, and the development of the PECARN rule, the optimal clinical prediction rule for the evaluation of children with possible CSI after blunt trauma must be determined. Understanding the diagnostic accuracy of CSI prediction rules among children will inform best practices to identify children at high risk for CSI who require imaging after blunt trauma. The objectives of this study were to determine and compare the diagnostic accuracy of 3 prospectively derived CSI clinical prediction rules (PECARN, NEXUS, CCR) in a cohort of pediatric trauma patients from a large, prospective observational study. We hypothesized that the PECARN CSI prediction rule will have superior diagnostic accuracy for identifying CSI in pediatric trauma patients and will result in a lower projected use of CT imaging for CSI evaluation.

## Methods

### Study Design and Setting

This was a planned secondary analysis from a large US prospective multicenter study to develop a clinical prediction rule for evaluating pediatric CSI. Details of the study methodology have been previously published.^3^ We enrolled children ages 0 to 17 years who experienced blunt trauma and presented to any of the 18 PECARN ED study sites from December 2018 to December 2021. All sites were pediatric level 1 trauma centers as designated by the American College of Surgeons or the State’s equivalent. Recruitment occurred in 2 phases with 9 sites each enrolling for 20 to 22 months. The primary outcome of the main CSI prediction rule study was the presence of CSI. CSIs were defined as any injury involving the cervical region of the spine (occiput to the seventh cervical vertebra, including ligaments attaching the seventh vertebrae to the first thoracic vertebra) demonstrated on any c-spine imaging modality, including radiography, skeletal survey, CT scan, and/or MRI. CSIs included vertebral fracture, ligamentous injury, intraspinal hemorrhage, or spinal cord injury (visualized on MRI or determined clinically as spinal cord injury without radiologic abnormality). CSI status was determined by masked review of hospital records 21 to 28 days postencounter. Uncertain diagnoses were verified with treating surgeons.^3^

This study followed the Strengthening the Reporting of Observational Studies in Epidemiology (STROBE) reporting guidelines for observational research. The study was approved using the single institutional review board process for all participating sites through the University of Utah. We had a waiver of informed consent for prospective observational data collection and medical record review, and obtained verbal consent for mail notification for parental telephone follow-up. This study was registered with ClinicalTrials.gov (NCT05049330).

### Selection of Participants

Eligibility for the primary study included children aged 0 to 17 years with known or suspected blunt trauma who were: (1) presenting from the scene of injury by emergency medical services; (2) receiving trauma team evaluation at the enrolling PECARN ED; and/or (3) c-spine imaging ordered as part of the ED evaluation. Children were excluded if they presented only with penetrating trauma.

### Measurements

We collected data on CSI risk factors for the patient from the ED treating physician (attending or pediatric emergency medicine fellow) on a structured data collection form, which included: injury mechanisms, predisposing medical conditions, history of neck and neurologic complaints prior to ED presentation or in the ED, and physical examination findings. From the electronic health record (EHR) we collected the patient’s demographic data and information on clinical management including if the patient was transferred to the PECARN ED from another facility, c-spine imaging obtained (ie, radiography, CT, MRI), spine surgeon consultation notes, and disposition from the ED. To determine if any CSI diagnoses were missed in the study population, the EHR was also reviewed to determine if any c-spine imaging was obtained 21 to 28 days after the initial ED encounter. If no imaging was obtained at the initial ED encounter and there was no record of additional c-spine imaging on EHR review, the patient’s guardian was contacted by phone 21 to 28 days after the initial ED visit to ascertain if CSI was diagnosed subsequently in another clinical setting.

### Outcomes

The primary outcome of this study was the diagnostic accuracy based on their test characteristics for identifying CSI among this cohort of pediatric patients with blunt trauma for each of the 3 prospectively derived CSI clinical prediction rules: PECARN, NEXUS, and CCR (Box). Secondary outcomes included projected imaging rates (radiography or CT, as appropriate) based on application of criteria of the 3 rules to the PECARN study cohort for clinically cleared, radiography, and CT imaging.

Box. **Criteria for PECARN CSI, NEXUS and CCR Prediction Rules**

**Pediatric Emergency Care Applied Research Network (PECARN) CSI Rule^3^**

- High risk factors—consider CT
  - GCS 3-8 or Unresponsive on AVPU scale
  - Abnormal airway, breathing or circulation
  - Focal neurologic deficit on examination
- CART-derived risk factors—consider radiography
  - Altered mental status^a^
  - Self-reported neck pain or neck tenderness on examination
  - Substantial head or torso injury^b^

**National Emergency X-Radiography Utilization Study (NEXUS)^15^**

- C-spine imaging recommended unless all criteria present:
  - No posterior midline c-spine tenderness
  - No evidence of intoxication
  - Normal level of alertness
    - GCS = 15
    - Ability to remember 3 objects in 5 min
    - No delayed response
  - No focal neurological deficits on motor or sensory examination
  - No painful distracting injuries

**Canadian C-spine Rule (CCR)^14^**

- For patients with GCS = 15 and normal vital signs with concern for c-spine injury
- High-risk criteria → radiology
  - Age ≥65 y
  - Dangerous mechanism^c^
  - Paresthesias in extremities
- Any low-risk criteria to safely examine neck range of motion?
  - Simple rear-end motor vehicle crash
  - Able to sit in the ED
  - Ambulatory at any time
  - Delayed onset of neck pain
  - Absent midline c-spine tenderness
  - If no to any of the above → radiology
- If yes to all above and then able to actively rotate neck laterally 45° → no radiology

^a^
Altered mental status was defined as GCS score of 9-14; verbal or pain on AVPU; or other signs of altered mental status.

^b^
Substantial injuries were defined as those that warranted inpatient observation or surgical intervention.

^c^
Dangerous mechanism was defined as: fall from ≥1 m/5 stairs; axial load to head; motor vehicle crash high speed (>100 km/h), rollover, ejection; motorized recreational vehicles; or bicycle collision.

### Statistical Analysis

We calculated descriptive frequencies for patient and clinical management characteristics. For each predictor in the 3 CSI clinical prediction rules, we reported the descriptive frequencies for the overall study population and for those aged 0 to 8 years and those 9 to 17 years old. Age was dichotomized into these groups as children younger compared with older than 9 years have anatomic differences increasing their risk and type of CSI.^8^ For the analyses of our primary outcome, we calculated the test characteristics (sensitivity, specificity, negative predictive value [NPV], and positive predictive value [PPV]) for each of the 3 rules for the overall cohort and for each age group.

To determine predicted imaging rates for each of the 3 CSI clinical prediction rules, we applied the criteria for each of the rules to this study cohort. For variables from this study cohort that did not exactly match the NEXUS and CCR variables, the study team chose those which most closely aligned with those in these rules (eTable 1 in Supplement 1). The outcome of clinically cleared was calculated based on the absence of any risk factor for CSI for all ages and then divided by age group (0 to 8 years, 9 to 17 years). For the PECARN rule, we calculated the radiography imaging rate based on the presence of any of the low-risk criteria and CT imaging rate based on the presence of any high-risk criteria in the study cohort (Box).^3^ As the NEXUS rule and CCR do not risk stratify to guide imaging modality type, predicted imaging rates for radiography and CT were obtained by applying the actual ratio of radiography and CT among those imaged within the PECARN C-spine cohort to the participants who had any risk factor for the NEXUS and CCR (eTable 1 in Supplement 1). All analyses were conducted in SAS version 9.4 (SAS Institute Inc). Significance was determined by 95% CIs.

## Results

There were 22 430 eligible children with blunt trauma enrolled in the primary study (eFigure 1 in Supplement 1). The median (IQR) age of enrolled children was 8.0 (2.0-13.0) years and 13 068 (53.8%) were male (Table 1). The most common mechanism of injury was fall (7444 [33.2%]) followed by motor vehicle crash (6358 [28.3%]). Among the children enrolled in the study 12 768 (56.9%) had c-spine imaging and 433 (1.9%) had a CSI. Of the 12 768 children who had any neck imaging, 8912 (69.8%) had radiography, and 3856 (30.2%) had CTs performed.

**Table 1.** Demographic Characteristics of Study Participants

Characteristics	Children, No. (%)		
	Overall (n = 22 430)	Age 0-8 y (n = 11 633)	Age 9-17 y (n = 10 797)
Demographics			
Age, median (IQR), y	8.0 (2.0-13.0)	3.0 (0.0-6.0)	13.0 (11.0-15.0)
Sex			
Female	9362 (41.7)	4878 (41.9)	4484 (41.5)
Male	13 068 (58.3)	6755 (58.1)	6313 (58.5)
Mechanism of injury			
Motor vehicle crash (driver or passenger)	6358 (28.3)	2948 (25.3)	3410 (31.6)
Motorcycle, ATV, motorized scooter crash	1250 (5.6)	269 (2.3)	981 (9.1)
Hit by car or other motor (pedestrian, cyclist, other)	1455 (6.5)	589 (5.1)	866 (8.0)
Fall	7444 (33.2)	5054 (43.4)	2390 (22.1)
Diving	38 (0.2)	4 (<0.1)	34 (0.3)
Sports or recreation related	2219 (9.9)	340 (2.9)	1879 (17.4)
Suspected child abuse	1327 (5.9)	1246 (10.7)	81 (0.8)
Assault or altercation	535 (2.4)	51 (0.4)	484 (4.5)
Other mechanism	1231 (5.5)	620 (5.3)	611 (5.7)
Unknown mechanism	573 (2.6)	512 (4.4)	61 (0.6)
Clinical course			
Any predisposing condition for CSI	144 (0.6)	62 (0.5)	82 (0.8)
Observed imaging rates			
No imaging	9570 (42.7)	5018 (43.1)	4552 (42.2)
Neck radiography	8912 (39.7)	5206 (44.8)	3706 (34.3)
C-spine computed tomography	3856 (17.2)	1380 (11.9)	2476 (22.9)
MRI only	76 (0.3)	23 (0.2)	53 (0.5)
Other imaging^a^	16 (0.1)	6 (0.1)	10 (0.1)
Transferred to your facility with cervical spine imaging	1656 (7.4)	706 (6.1)	950 (8.8)
ED disposition^b^			
Home	14 948 (66.6)	7633 (65.6)	7315 (67.8)
Intensive care unit	1614 (7.2)	898 (7.7)	716 (6.6)
Admission to inpatient unit	5038 (22.5)	2717 (23.4)	2321 (21.5)
Operating room	618 (2.8)	312 (2.7)	306 (2.8)
Death in the emergency department	43 (0.2)	32 (0.3)	11 (0.1)
Other	169 (0.8)	41 (0.4)	128 (1.2)

Abbreviations: ATV, all-terrain vehicle; CSI, cervical spine injury; CT, computed tomography; ED, emergency department; MRI, magnetic resonance imaging.

^a^ This category includes other imaging modalities that reported information regarding the cervical spine including chest radiography, head CT, and chest CT.

^b^ Admission to inpatient unit includes to general inpatient and short stay/observation unit (<24 hours). Other includes transferred to another facility, left prior to being discharged or other (selected on data collection form).

### Presence of CSI Prediction Rule Factors

For the PECARN CSI rule, 9225 children (41.1%) had the presence of at least 1 CSI risk factor (eTable 2 in Supplement 1). Self-reported neck pain (4711 [21.0%]) and neck pain upon examination (3706 [16.5%]) were most commonly present. Altered mental status was reported among 2169 patients (9.7%). Overall, there was low prevalence of the high-risk factors: GCS 3 to 8 (522 [2.3%]); unresponsiveness (340 [1.5%]); abnormal airway, breathing, or circulation (982 [4.4%]); or focal neurologic deficits (605 [2.7%]) with similar proportions in both age groups. However, older (ie, ages 9 to 17 years) compared with younger (0 to 8 years) children had a higher prevalence of self-reported neck pain (3471 of 10 797 [32.1%] vs 1240 of 11 633 [10.7%]) and neck pain upon examination (2784 of 10 797 [25.8%] vs 922 of 11 633 [7.9%]). In contrast, younger children had a higher prevalence of signs of substantial head injury (1602 of 11 633 [13.8%] vs 811 of 10 797 [7.5%]). The percentage of children receiving c-spine imaging closely corresponded with the presence of the CSI risk factors (eFigure 2 in Supplement 1).

### Comparison of CSI Prediction Rules

Overall, the PECARN CSI rule demonstrated the best test characteristics with a sensitivity for detection of CSI of 93.3% (95% CI, 90.9%-95.7%), followed by CCR (90.8%; 95% CI, 88.0%-93.5%) and NEXUS (85.7%; 95% CI, 82.4%-89.0%) (Table 2; Figure, panel A). The PECARN CSI rule also had the highest sensitivity in both age groups (age 0 to 8 years, 92.7%; 95% CI, 89.1%-96.4%; age 9 to 17 years, 93.8%; 95% CI, 90.7%-96.8%). For specificity NEXUS (65.3%, 95% CI 64.6%-65.9%) was higher than the PECARN CSI (59.9%; 95% CI, 59.3%-60.5%) and CCR (57.1%; 95% CI, 56.4%-57.8%) rules (Table 2; Figure, panel B). The NPVs were similar although the PECARN CSI rule was highest at 99.8% (95% CI, 99.7%-99.9%), followed by CCR (99.7%; 95% CI, 99.6%-99.8%) and NEXUS (99.6%; 95% CI, 99.5%-99.7%) (Table 2). The AUCs for the ROCs were PECARN CSI rule (0.77), NEXUS (0.75), and CCR (0.74) (eFigure 3 in Supplement 1). The AUC was similar for children aged 0 to 8 years and 9 to 17 years (eFigure 4 and 5 in Supplement 1).

**Table 2.** Comparison of Diagnostic Accuracy and Test Characteristics of the PECARN, NEXUS, and CCR CSI Decision Rules

Decision rule	CSI identified, No.				Test characteristics, % (95% CI)			
	True positive	False positive	False negative	True negative	Sensitivity	Specificity	NPV	PPV
**Overall**								
PECARN CSI rule^a^	404	8821	29	13 176	93.3 (90.9-95.7)	59.9 (59.3-60.5)	99.8 (99.7-99.9)	4.4 (4.0-4.8)
NEXUS^b^	371	7639	62	14 358	85.7 (82.4-89.0)	65.3 (64.6-65.9)	99.6 (99.5-99.7)	4.6 (4.2-5.1)
CCR^c^	393	9436	40	12 561	90.8 (88.0-93.5)	57.1 (56.4-57.8)	99.7 (99.6-99.8)	4.0 (3.6-4.4)
**Age 0-8 y**								
PECARN CSI rule	179	3675	14	7765	92.7 (89.1-96.4)	67.9 (67.0-68.7)	99.8 (99.7-99.9)	4.6 (4.0-5.3)
NEXUS	169	3210	24	8230	87.6 (82.9-92.2)	71.9 (71.1-72.8)	99.7 (99.6-99.8)	5.0 (4.3-5.7)
CCR	168	3568	25	7872	87.0 (82.3-91.8)	68.8 (68.0-69.7)	99.7 (99.6-99.8)	4.5 (3.8-5.2)
**Age 9-17 y**								
PECARN CSI rule	225	5146	15	5411	93.8 (90.7-96.8)	51.3 (50.3-52.2)	99.7 (99.6-99.9)	4.2 (3.7-4.7)
NEXUS	202	4429	38	6128	84.2 (79.5-88.8)	58.0 (57.1-59.0)	99.4 (99.2-99.6)	4.4 (3.8-5)
CCR	225	5868	15	4689	93.8 (90.7-96.8)	44.4 (43.5-45.4)	99.7 (99.5-99.8)	3.7 (3.2-4.2)

Abbreviations: CCR, Canadian Cervical Spine (c-spine) rule; CSI, cervical spine injury; NEXUS, National Emergency X-Radiography Utilization Study; PECARN, Pediatric Emergency Care Applied Research Network for cervical spine injury.

^a^ With application of the PECARN CSI rule there would have been 29 with missed CSI: 19 required further management (19 collar).

^b^ With application of the NEXUS rule there would have been 62 patients with missed CSI: 49 required further management (42 collar, 1 Minerva brace, 7 surgical).

^c^ With application of the CCR, there would have been 40 patients with missed CSI: 24 required further management (22 collar, 1 Halo, 1 surgical).

**Figure.**
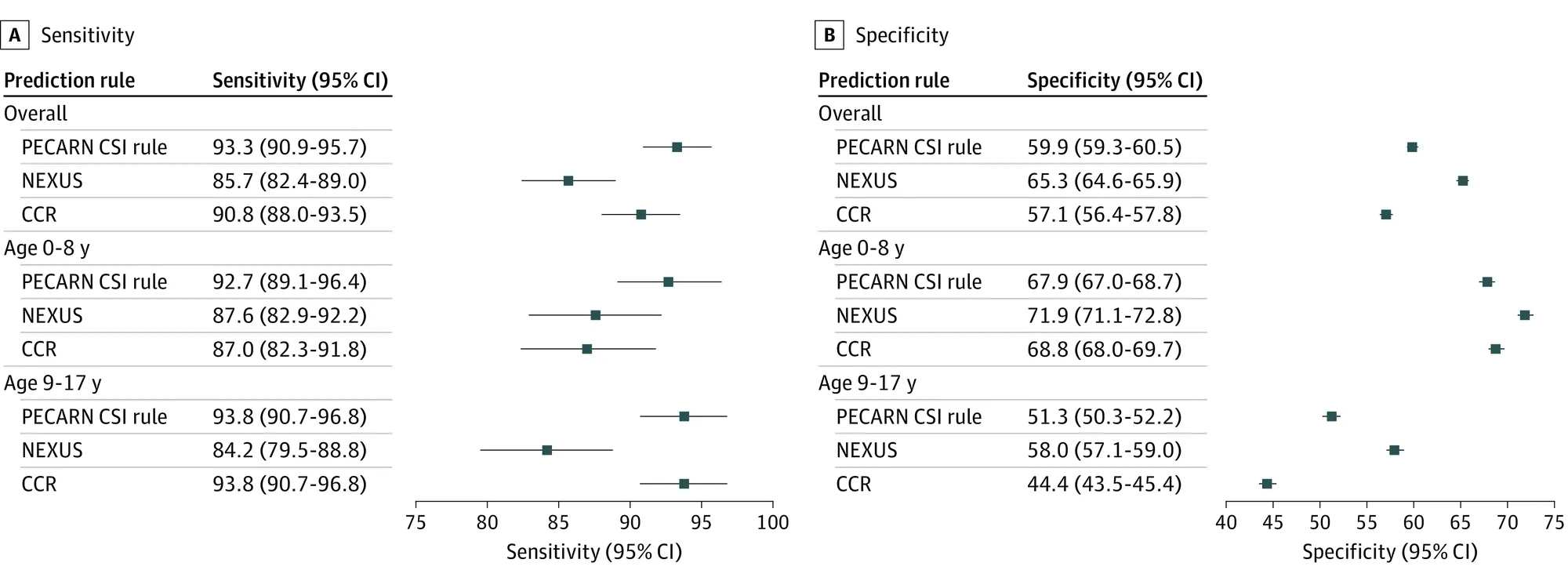
Forest Plot for the PECARN CSI, NEXUS, and CCR Prediction Rules CCR indicates Canadian Cervical Spine (c-spine) rule; NEXUS, National Emergency X-Radiography Utilization Study; PECARN CSI, Pediatric Emergency Care Applied Research Network for cervical spine injury.

### Predicted Imaging Rates

Strictly applying each rule resulted in projected imaging frequencies (radiography and CT combined) of 41.1% (9225 children) for PECARN, 35.7% (8010 children) for NEXUS, and 43.8% (9829 children) for CCR (Table 3). However, when comparing the 3 rules, the NEXUS had the lowest predicted radiography imaging rate of 24.9% (5591 children), followed by CCR at 30.6% (6861 children). When considering the imaging rate for CT, the PECARN rule had the lowest predicted rate of 6.9% (1549 children), followed by the NEXUS rule at 10.8% (2419 children). The 3 different rules demonstrated similar patterns between the 2 different age groups.

**Table 3.** Comparison of Imaging Rates With Application of the PECARN CSI, NEXUS, and CCR Prediction Rules

Decision rule	Children, No. (%)		
	Clinically cleared^a^	Radiography imaging rate^b^	Predicted CT imaging rate^c^
**All ages (0-17 y)**			
Actual^d^	9662 (43.1)	8912 (39.7)	3856 (17.2)
Predicted			
PECARN CSI rule	13 205 (58.9)	7676 (34.2)	1549 (6.9)
NEXUS	14 420 (64.3)	5591 (24.9)	2419 (10.8)
CCR	12 601 (56.2)	6861 (30.6)	2968 (13.2)
**Age 0-8 y**			
Actual^d^	5047 (43.4)	5206 (44.8)	1380 (11.9)
Predicted			
PECARN CSI rule	7779 (66.9)	3163 (27.2)	691 (5.9)
NEXUS	8254 (71.0)	2671 (23.0)	708 (6.1)
CCR	7897 (67.9)	2953 (25.4)	783 (6.7)
**Age 9-17 y**			
Actual^d^	4615 (42.7)	3706 (34.3)	2476 (22.9)
Predicted			
PECARN CSI rule	5426 (50.3)	4513 (41.8)	858 (7.9)
NEXUS	6166 (57.1)	2776 (25.7)	1855 (17.2)
CCR	4704 (43.6)	3653 (38.8)	2440 (22.6)

Abbreviations: CCR, Canadian Cervical Spine (c-spine) rule; CSI, cervical spine injury; NEXUS, National Emergency X-Radiography Utilization Study; PECARN, Pediatric Emergency Care Applied Research Network for cervical spine injury.

^a^ Patients were considered clinically cleared if they had no rule risk factors.

^b^ For PECARN CSI rule, NEXUS and CCR this was calculated by applying the proportion of PECARN CSI study participants who had a radiography performed to study participants with any risk factor for these rules.

^c^ For PECARN CSI rule, NEXUS and CCR this was calculated by applying the proportion of PECARN CSI study participants who had a CT performed to study participants with any risk factor for these rules.

^d^ Actual number of participants from the PECARN CSI rule study in each of these categories.

## Discussion

In this large, prospective, observational multicenter study of children with blunt trauma, we demonstrated the PECARN CSI rule has superior test characteristics for identifying children with CSI compared with the NEXUS and CCR rules. The PECARN CSI rule had the highest sensitivity for the detection of CSI among the entire age group of patients and when the patients were divided into younger (0 to 8 years) and older (9 to 17 years) age groups. It also had the highest AUC compared with the NEXUS and CCR rules. With the modified application of the NEXUS and CCR rules, PECARN is predicted to have the lowest CT imaging rates. Application of the PECARN CSI rule demonstrated the lowest number of missed CSIs compared with NEXUS and CCR, which demonstrated more children with missed CSIs, including those requiring surgical interventions.

Although CSIs rarely occur after blunt trauma, they can result in lifelong neurologic disability and even death.^1,2,3,4^ Children younger than age 8 years compared with older children and teenagers, have unique anatomic features, increasing their risk for cranio-cervical junction and upper cervical spine injuries as well as ligamentous injuries, including spinal cord injury without radiologic abnormalities.^8,18^ Advanced imaging (ie, CT, MRI) is the criterion standard for diagnosing CSI.^1,6^ Increasing use of c-spine CT and MRI imaging among children presenting with trauma from 2010 to 2019, without a corresponding linear increase in CSI diagnoses, has been reported in 1 study among 33 US children’s hospitals.^4^ Without a highly accurate rule, there is wide variability in the protocols used for pediatric cervical spine clearance after blunt trauma.^19^

Judicious use of these modalities is needed due to cost and, for CT specifically, the increased lifetime risk of radiation-induced cancer.^9,10^ Therefore, determining the optimal stratified approach for using radiologic imaging in the evaluation of CSI for children presenting with blunt trauma is needed to provide appropriate evidence-based and equitable care. The stratified approach of the highly sensitive PECARN rule provides a guideline based on risk of CSI for the use of radiography vs CT. This strategy promotes the accurate diagnosis of CSI, while limiting CT, and the associated exposure to ionizing radiation, to those at higher risk for CSI.

A Cochrane Systematic Review, published before the PECARN rule, to determine the diagnostic accuracy of c-spine clinical prediction rules for pediatric patients evaluated for CSI after blunt trauma included 5 studies eligible for analysis. This review, which included analysis of the NEXUS and CCR prediction rules, reported there was not enough evidence to determine which CSI prediction rules were most effective for detecting CSIs in children, especially those younger than 8 years old. They concluded application of these rules could potentially result in unnecessary CT imaging for children without CSI.^1^

A few pediatric specific studies have identified factors associated with CSI in children after blunt trauma. A retrospective, multicenter retrospective study of children less than 3 years old developed a multinomial model using clinical criteria and a predictive model to risk stratify children for osseous and ligamentous injury.^20^ One case-control study of 540 children under 16 years old with CSI identified 8 factors associated with CSI: altered mental status, focal neurologic findings, neck pain, torticollis, substantial torso injury, conditions predisposing to cervical spine injury, diving, and high-risk motor vehicle crash.^21^ A multicenter, prospective study developed a de novo model from a cohort of 4091 children 0 to 17 years old with blunt trauma and included the factors of diving, axial load, neck pain, inability to move the neck, altered mental status, intubation, or respiratory distress.^2^ The case-control study and the de novo model informed the development of the current PECARN CSI prediction rule study,^3^ which has improved sensitivity compared with these earlier pediatric studies and the NEXUS and CCR prediction rules.

In the analysis of projected imaging frequencies for each of the prediction rules applied to this study sample, the PECARN CSI rule and the CCR demonstrated similar overall imaging rates, while the NEXUS rule had the lowest overall predicted imaging rate. However, these results must be interpreted with the understanding that not all data collected for the PECARN CSI study aligned with variables in the NEXUS and CCR prediction rules. Also, these rules were not developed with the approach of risk stratification for the imaging modalities of radiography and CT. Additionally, comorbid injuries in the PECARN CSI study were defined as substantial head or torso injuries warranting hospitalization or surgical intervention and did not include long bone injuries.^3^ In contrast, NEXUS uses the broad variable for comorbid injuries, “painful distracting injuries,”^15,22,23^ defined as “a clinically apparent, painful injury that might distract them from the pain of a cervical-spine injury.”^15,22^ Thus, for example, a patient with a femur fracture would be considered to have a painful distracting injury, which could result in c-spine imaging based on the NEXUS rule. However, the PECARN rule variable “substantial torso or head injury” was adapted to the NEXUS rule for the projected imaging rates. Using this definition, children with a femur fracture would not have been included in the study cohort and would not be captured for application to the NEXUS rule for imaging prediction. It is possible that the NEXUS rule and CCR would have demonstrated different results for the rule performance if all the variables matched exactly. Given the specific variables in these prediction rules were primarily focused on low risk factors it is unlikely the overall test characteristics would have demonstrated improved performance among this study population.

### Limitations

This study has limitations. Our study population included patients presenting after trauma to one of the participating tertiary care pediatric trauma center PECARN ED study sites; therefore, our results may not be generalizable to other EDs or clinical settings. It is also possible that CSI diagnoses were missed; however, radiology records were reviewed for patients who may have returned to the index hospital after discharge and follow-up phone calls were conducted for patients who did not have a subsequent visit to the index hospital. Finally, the NEXUS rule and CCR as originally derived do not specifically include when to image with CT for CSI. Calculations of projected imaging rates and rules comparisons were based on our mapping of the variables in our study cohort to the NEXUS rule and CCR. Therefore, our interpretation of these rules to determine missed CSIs and projected imaging rates by risk stratification could vary if these variables are mapped using a different method as well as if our study cohort variables exactly matched the variables in these rules.

## Conclusions

The PECARN CSI prediction rule had the highest sensitivity and NPV for identifying children at low risk for CSI after blunt trauma. It also demonstrated the lowest projected CT imaging rate among our study population. Widespread implementation and study of the PECARN CSI rule in general and community hospitals will be essential to better understand the rule performance among a generalizable pediatric trauma population. Future work will be essential to understand the PECARN CSI prediction rule’s effects on advanced imaging rates for CSI overall and in providing optimal pediatric trauma care.

## Notes

Supplement 1. eFigure 1. Flow Diagram of Patient Enrollment for PECARN CSI Prediction Rule Study eFigure 2. Prevalence of Clinical Rule Predictors and Imaging for the PECARN, CCR, and NEXUS CSI Prediction Rules eFigure 3. Receiver Operator Curves of the PECARN, CCR, and NEXUS CSI Prediction Rules eFigure 4. Receiver Operator Curves of the PECARN CSI, CCR, and NEXUS Prediction Rules for participants of age 0 - 8 years eFigure 5. Receiver Operator Curves of the PECARN CSI, CCR, and NEXUS Prediction Rules for participants of age 9 - 17 years

Supplement 2. Nonauthor Collaborators

Supplement 3. Data Sharing Statement
