# Supplementary material for: Comparison of Cervical Spine Injury Clinical Prediction Rules for Children After Blunt Trauma
Source: JAMA Netw Open. 2025 Dec 19;8(12):e2549403. doi: 10.1001/jamanetworkopen.2025.49403 (PMC12717614; doi:10.1001/jamanetworkopen.2025.49403)
Supplement: Supplement 1. — eFigure 1. Flow Diagram of Patient Enrollment for PECARN CSI Prediction Rule Study eFigure 2. Prevalence of Clinical Rule Predictors and Imaging for the PECARN, CCR, and NEXUS CSI Prediction Rules eFigure 3. Receiver Operator Curves of the PECARN, CCR, and NEXUS CSI Prediction Rules eFigure 4. Receiver Operator Curves of the PECARN CSI, CCR, and NEXUS Prediction Rules for participants of age 0 - 8 years eFigure 5. Receiver Operator Curves of the PECARN CSI, CCR, and NEXUS Prediction Rules for participants of age 9 - 17 years [file jamanetwopen-e2549403-s001.pdf]

Supplemental Online Content

Lee LK, Ahmad F, Browne LR, et al. Comparison of C-spine injury clinical prediction rules for children after blunt trauma. *JAMA Netw Open.* 2025;8(12):e2549403. doi:10.1001/jamanetworkopen.2025.49403

eFigure 1. Flow Diagram of Patient Enrollment for PECARN CSI Prediction Rule Study

eFigure 2. Prevalence of Clinical Rule Predictors and Imaging for the PECARN, CCR, and NEXUS CSI Prediction Rules

eFigure 3. Receiver Operator Curves of the PECARN, CCR, and NEXUS CSI Prediction Rules

eFigure 4. Receiver Operator Curves of the PECARN CSI, CCR, and NEXUS Prediction Rules for participants of age 0 - 8 years

eFigure 5. Receiver Operator Curves of the PECARN CSI, CCR, and NEXUS Prediction Rules for participants of age 9 - 17 years

This supplemental material has been provided by the authors to give readers additional information about their work.

© 2025 Lee LK et al. *JAMA Network Open*.

eTable 1. Variables Applied from PECARN CSI Study Cohort and Applied to NEXUS and CCR Prediction Rules Factors

Original Rule Adaptation	Variables used from PECARN CSI Study
**NEXUS**[12]	
**Risk factors**	
• Abnormal level of alertness, GCS < 15	Altered mental status, Glasgow Coma Score < 15
• Intoxication	Suspicion for alcohol or drug intoxication
• Focal neurologic deficits	Examination focal neurological
• Posterior midline c-spine tenderness	Neck pain upon examination
• Painful distracting injury	Substantial Torso OR Head Injury
• Inability to remember 3 objects in 5 minutes	(variable not available)
• Delayed response	(variable not available)
**CCR**[13]	
**Risk factors**	
• GCS< 15 and unstable trauma patient with concern for c-spine injury*	GCS < 15, Abnormal airway, breathing, or circulation
• Dangerous trauma mechanism	
○ Fall ≥ 3 feet or five stairs	High risk fall
○ Axial load to the head (e.g. diving)	Axial load or diving
○ Motor vehicle crash >100 km/hour, rollover or ejection from vehicle	High risk motor vehicle crash
○ Motor vehicle crash involving recreational vehicle or bicycle collision	Motorcycle/ATV/motorized scooter/bicycle crash, etc.
• Paresthesias in extremities	Paresthesias (abnormal tactile sensation) (on exam or self-report)
• Inability to actively rotate neck 45° left and right	Inability to move neck upon examination
• Known vertebral disease[3]	Known vertebral disease or previous c-spine injury
**Low-risk factors allowing safe assessment of neck range of motion**	
• Simple rear-end motor vehicle crash	Not a high risk motor vehicle crash
• Absent midline c-spine tenderness	Absence of neck pain upon examination
• Able to sit in the ED	(variable not available)
• Ambulatory at any time	(variable not available)
• Delayed onset of neck pain	(variable not available)
*CCR excluded patients with GCS < 15, abnormal vital signs, and known vertebral disease.	

© 2025 Lee LK et al. JAMA Network Open.

eTable 2. Prevalence of Risk Factors for the PECARN CSI, NEXUS, and CCR Prediction Rules among the PECARN CSI Study Cohort

		Age	
	Overall (N = 22,430)	0 – 8 years (N = 11,633)	9 – 17 years (N = 10,797)
PECARN CSI Rule[3]	9,225 (41.1%)	3,854 (33.1%)	5,371 (49.7%)
Glasgow Coma Score: 3-8	522 (2.3%)	295 (2.5%)	227 (2.1%)
AVPU:* U	340 (1.5%)	185 (1.6%)	155 (1.4%)
Abnormal airway, breathing, or circulation findings	982 (4.4%)	537 (4.6%)	445 (4.1%)
Focal Neurologic Deficits	605 (2.7%)	176 (1.5%)	429 (4.0%)
Altered mental status	2,169 (9.7%)	1,145 (9.8%)	1024 (9.5%)
Self reported neck pain	4,711 (21.0%)	1,240 (10.7%)	3,471 (32.1%)
Substantial torso injury	1,149 (5.1%)	389 (3.3%)	760 (7.0%)
Signs of substantial head injury	2,413 (10.8%)	1,602 (13.8%)	811 (7.5%)
Neck pain upon examination	3,706 (16.5%)	922 (7.9%)	2,784 (25.8%)
NEXUS rule[13]	8,010 (35.7%)	3,379 (29.0%)	4,631 (42.9%)
Altered mental status	2,169 (9.7%)	1,145 (9.8%)	1024 (9.5%)
Suspicion for alcohol or drug intoxication	196 (0.9%)	41 (0.4%)	155 (1.4%)
Focal Neurologic Deficits	605 (2.7%)	176 (1.5%)	429 (4.0%)
Substantial torso or head injury	3,365 (15.0%)	1,905 (16.4%)	1,460 (13.5%)
Neck pain upon examination	3,706 (16.5%)	922 (7.9%)	2,784 (25.8%)
Canadian C-spine rule (CCR)[14]	9,829 (43.8%)	3,736 (32.1%)	6,093 (56.4%)
Abnormal airway, breathing, or circulation findings	982 (4.4%)	537 (4.6%)	445 (4.1%)
Glasgow Coma Score: < 15	1,867 (8.3%)	1,025 (8.8%)	842 (7.8%)
Fall and fall height of greater than 10 feet	766 (3.4%)	521 (4.5%)	245 (2.3%)
Diving	38 (0.2%)	4 (0.0%)	34 (0.3%)
Axial Load	725 (3.2%)	309 (2.7%)	416 (3.9%)
MVC and Any one of the following: intrusion, ejection, death, telemetry	1,271 (5.7%)	578 (5.0%)	693 (6.4%)
Motorcycle/ATV/motorized scooter crash, etc.	1,250 (5.6%)	269 (2.3%)	981 (9.1%)
Struck while riding a bicycling	371 (1.7%)	84 (0.7%)	287 (2.7%)
Self reported Paresthesia (abnormal tactile sensation)	547 (2.4%)	47 (0.4%)	500 (4.6%)
Paresthesia (abnormal tactile sensation)	270 (1.2%)	24 (0.2%)	246 (2.3%)
Inability to move the neck upon examination	786 (3.5%)	330 (2.8%)	456 (4.2%)
Any predisposing condition for CSI	144 (0.6%)	62 (0.5%)	82 (0.8%)
Extremity weakness	321 (1.4%)	74 (0.6%)	247 (2.3%)
Self reported neck pain	4,711 (21.0%)	1,240 (10.7%)	3,471 (32.1%)
Neck pain upon examination	3,706 (16.5%)	922 (7.9%)	2,784 (25.8%)

*AVPU scale: Alert, Verbal, Pain, Unresponsive scale of consciousness

© 2025 Lee LK et al. JAMA Network Open.

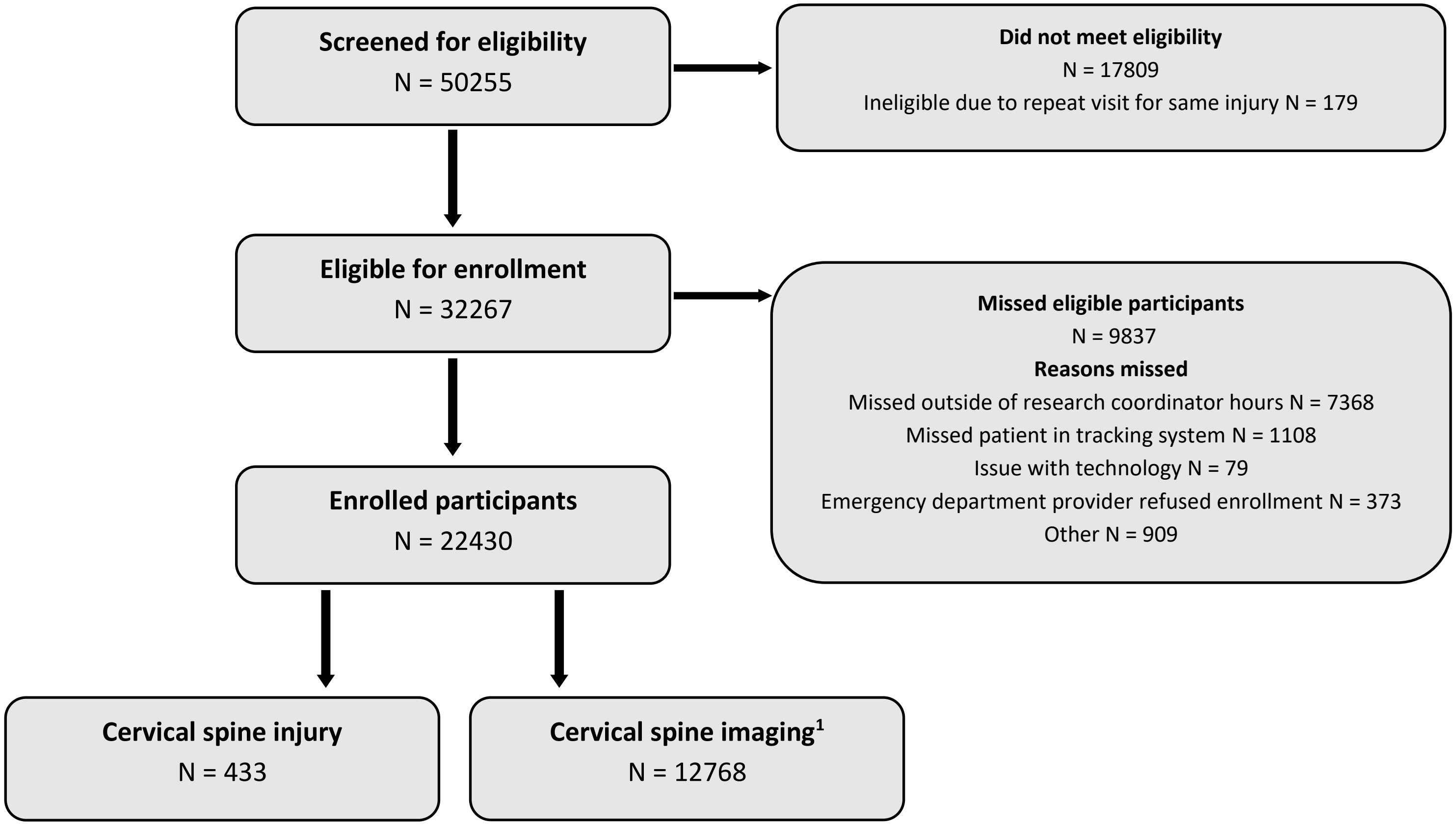

[1]Cervical spine imaging defined as any cervical spine X-ray, skeletal survey, cervical spine CT, or cervical spine MRI.

eFigure 1. Flow Diagram of Patient Enrollment for PECARN CSI Prediction Rule Study

© 2025 Lee LK et al. JAMA Network Open.

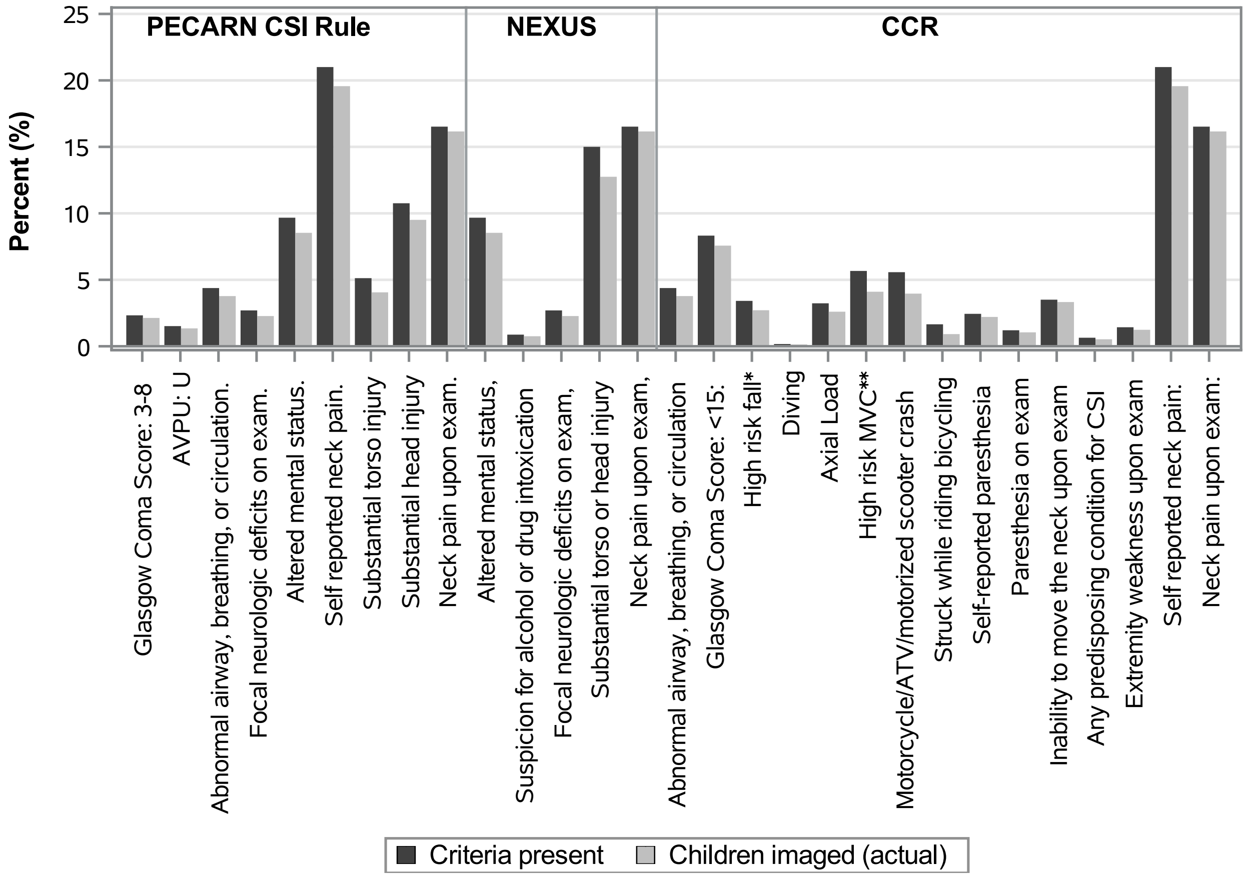

*High risk fall defined as fall with fall height of greater than 10 feet.
**High risk MVC defined as Motor vehicle crash, driver or passenger and any one of the following: intrusion, ejection, death, telemetry.

eFigure 2. Prevalence of Clinical Rule Predictors and Imaging for the PECARN, CCR, and NEXUS CSI Prediction Rules

© 2025 Lee LK et al. JAMA Network Open.

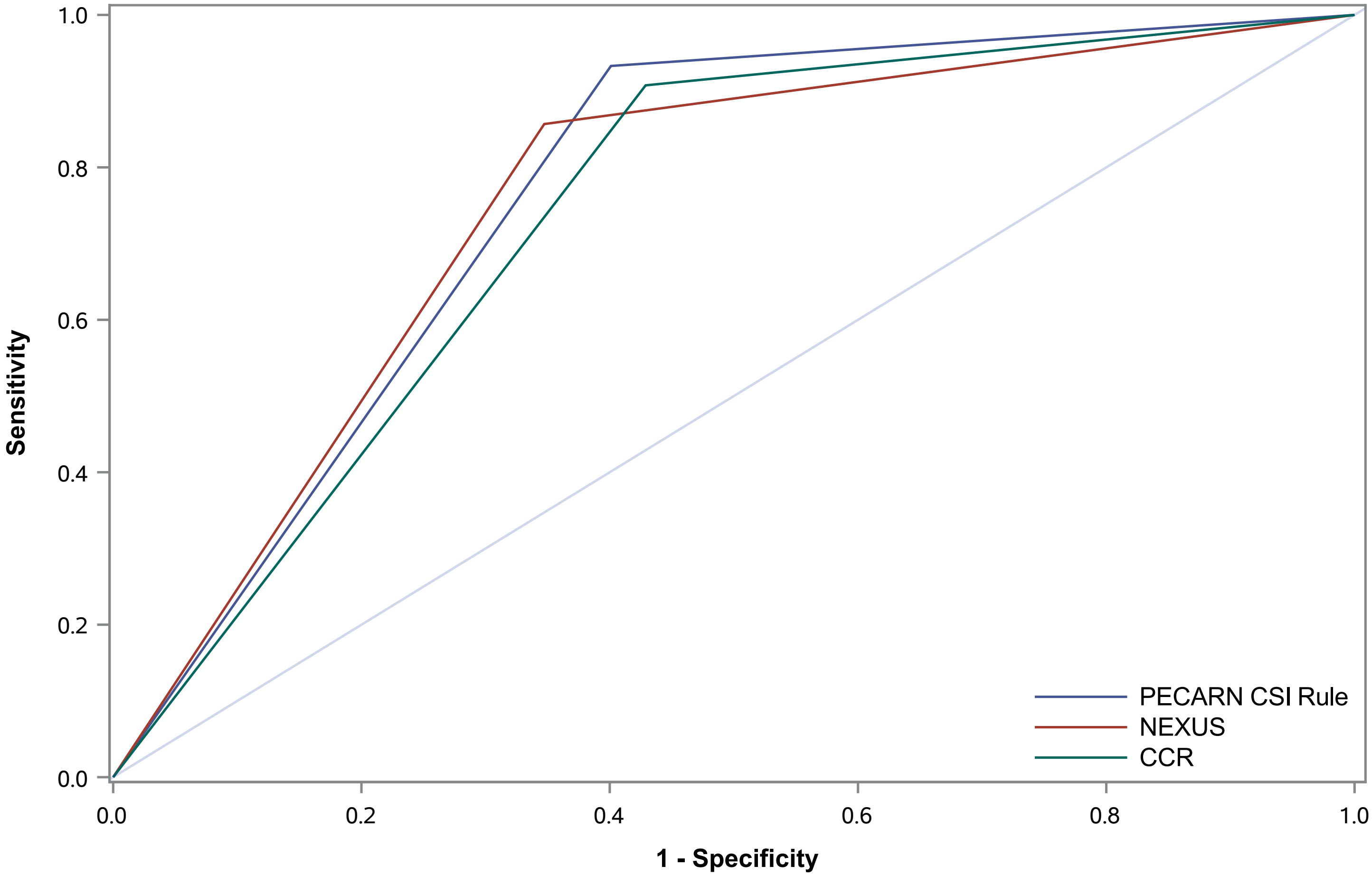

eFigure 3. Receiver Operator Curves of the PECARN CSI, CCR, and NEXUS Prediction Rules
Area under the curve (AUC): PECARN CSI rule 0.77, NEXUS rule 0.75, and Canadian C-spine rule (CCR) 0.74.

© 2025 Lee LK et al. JAMA Network Open.

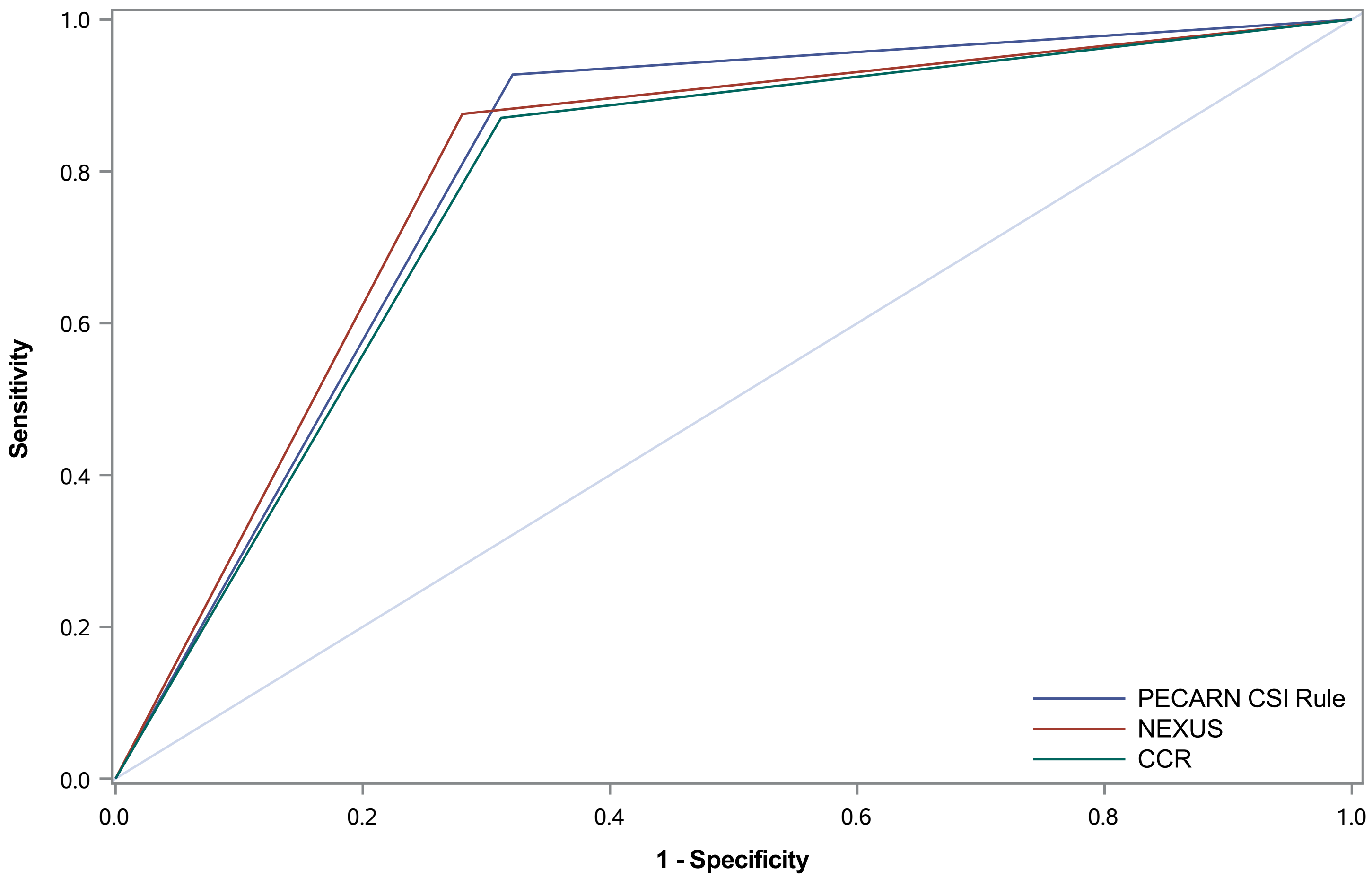

eFigure 4a. Receiver Operator Curves of the PECARN CSI, CCR, and NEXUS Prediction Rules for participants of age 0 - 8 years
Area under the curve (AUC): PECARN CSI rule 0.8, NEXUS rule 0.8, and Canadian C-spine rule (CCR) 0.78.

© 2025 Lee LK et al. JAMA Network Open.

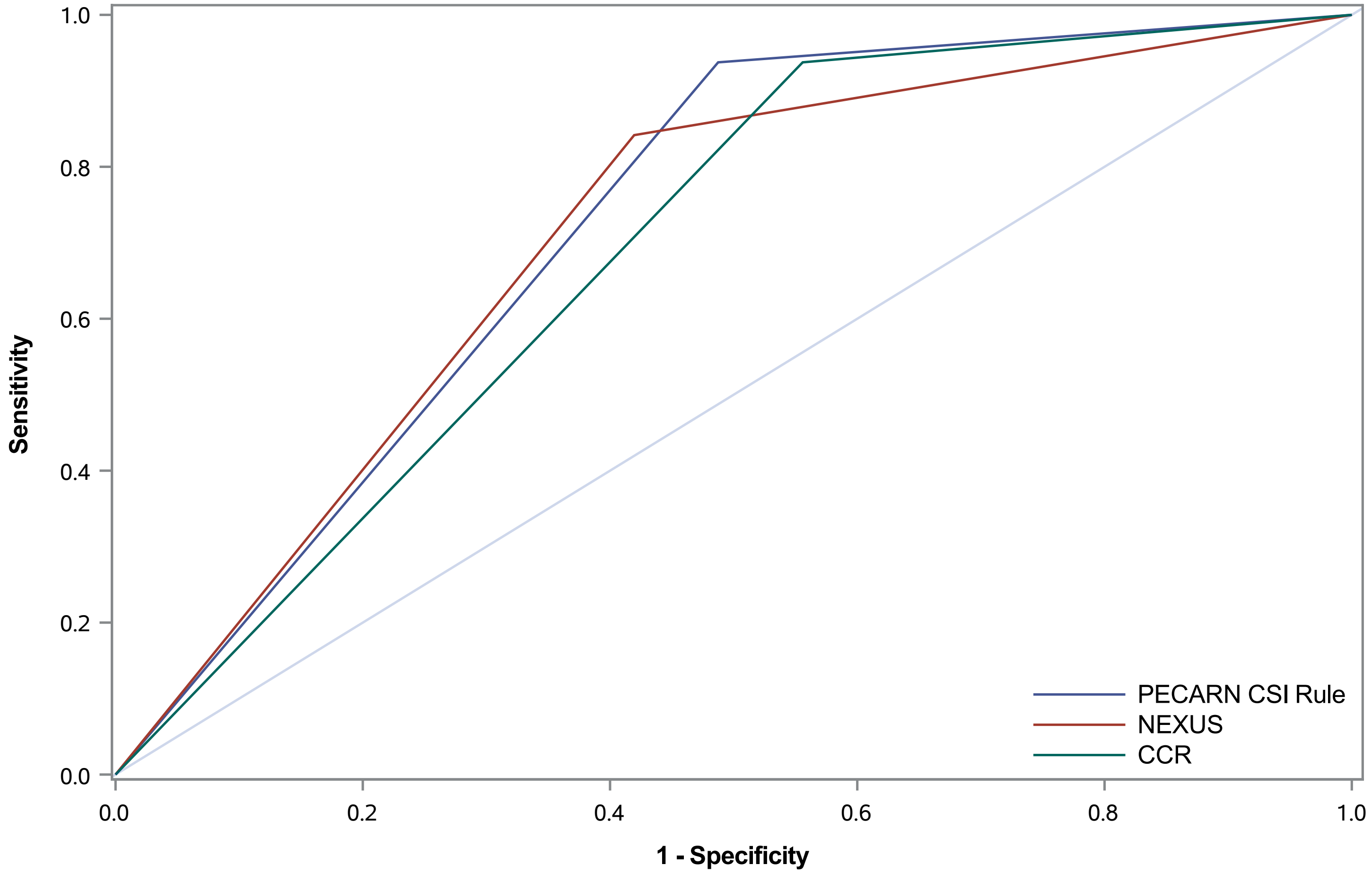

eFigure 4b. Receiver Operator Curves of the PECARN CSI, CCR, and NEXUS Prediction Rules for participants of age 9 - 17 years
Area under the curve (AUC): PECARN CSI rule 0.73, NEXUS rule 0.71, and Canadian C-spine rule (CCR) 0.69.

© 2025 Lee LK et al. JAMA Network Open.
