# Supplementary material for: Comparison of Cervical Spine Injury Clinical Prediction Rules for Children After Blunt Trauma
Source: JAMA Netw Open. 2025 Dec 19;8(12):e2549403. doi: 10.1001/jamanetworkopen.2025.49403 (PMC12717614; doi:10.1001/jamanetworkopen.2025.49403)
Supplement: Supplement 2. — Data Sharing Statement [file jamanetwopen-e2549403-s002.pdf]

Data Sharing Statement

Lee. Comparison of C-spine Injury Clinical Prediction Rules for Children After Blunt Trauma. *JAMA Netw Open*. Published December 12, 2025. doi:10.1001/jamanetworkopen.2025.49403

Data
Data available: Yes
Data types: Deidentified participant data, Data dictionary
How to access data: Request for data must be sent to Dr. Julie Leonard at Julie.Leonard@nationwidechildrens.org
When available: beginning date: 08-01-2029

Supporting Documents
Document types: None

Additional Information
Who can access the data: researchers whose proposed use of the data has been approved
Types of analyses: for a specified purpose
Mechanisms of data availability: after approval of a proposa with a signed data access agreement
